# Late effects of caffeine use on sleep of infants born prematurely

**DOI:** 10.1590/1984-0462/2024/42/2022224

**Published:** 2023-10-23

**Authors:** Ana Carolina Nunes de Oliveira, Ana Paula Cruz de Castro Leão, Ana Lucia Goulart, Allan Chiaratti de Oliveira, Vânia D'Almeida

**Affiliations:** a Universidade Federal de São Paulo, São Paulo, SP, Brasil.

**Keywords:** Sleep, Preterm infants, Caffeine, Obstructive sleep apnea, Sleep habits, Sono, Prematuro, Cafeína, Apneia obstrutiva do sono, Hábitos de sono

## Abstract

**Objective:**

This study aimed to evaluate whether the therapeutic use of caffeine for premature newborns is associated with changes in sleep habits and the presence of obstructive sleep apnea in childhood.

**Methods:**

This is a cross-sectional single-center study in which the caretakers of 87 children aged 5–10 years, born full-term or preterm, treated or not with caffeine in the neonatal period, answered questionnaires to screen for obstructive sleep apnea (Pediatric Obstructive Sleep Apnea Screening Tool [PosaST]) and to characterize the sleep habits (Children's Sleep Habits Questionnaire [CSHQ]) of their children. ANOVA and linear regression tests were performed to verify possible differences between the groups.

**Results:**

Children born prematurely who were treated with caffeine woke up significantly later on weekdays than those born at term (09h±00h58 and 07h43±1h15, respectively, p=0.022) and had longer total daily sleep time also compared to those born at term (10h24±1h08 and 09h29±1h08, respectively, p<0.001). There was no significant difference between the three groups in overall PosaST and CSHQ scores.

**Conclusions:**

Caffeine use in the neonatal period did not impair sleep habits later in life and did not lead to increased obstructive sleep apnea scores in prematurely born children compared to those born at term.

## RESUMO

**Objetivo:**

Avaliar se o uso terapêutico de cafeína para recém-nascidos prematuros se associa a alterações nos hábitos de sono e à presença de apneia obstrutiva do sono na infância.

**Métodos:**

Este é um estudo unicêntrico transversal no qual os responsáveis por 87 crianças com idades entre cinco e dez anos, nascidas a termo ou pré-termo e tratadas ou não com cafeína no período neonatal responderam a questionários para triagem de apneia obstrutiva do sono (*Pediatric Obstructive Sleep Apnea Screening Tool* – PosaST) e para a caracterização dos hábitos de sono (*Children's Sleep Habits Questionnaire* – CSHQ) de seus filhos. Foram realizados testes de variância (ANOVA) e de regressão linear para verificar possíveis diferenças entre os grupos.

**Resultados:**

As crianças nascidas prematuras que foram tratadas com cafeína acordaram significativamente mais tarde nos dias de semana do que as nascidas a termo (09h±00h58 e 07h43±1h15, respectivamente, p=0,022) e tiveram maior tempo total de sono diário também comparadas às nascidas a termo (10h24±1h08 e 09h29±1h08, respectivamente, p<0,001). Não houve diferença significativa entre os três grupos na pontuação geral dos questionários PosaST e CSHQ.

**Conclusões:**

O uso de cafeína no período neonatal não prejudicou tardiamente os hábitos de sono e não levou ao aumento dos escores de apneia obstrutiva do sono de crianças nascidas prematuras quando comparadas com crianças nascidas a termo.

## INTRODUCTION

Brazil ranks 10th among the countries with the highest frequency of premature births, with 11.7% of all live births.^1^ Furthermore, prematurity is the leading cause of mortality in the first 5 years of life and is associated with high morbidity for survivors.^2^

Premature newborns present high respiratory morbidity and mortality. Apnea of prematurity is a self-limited morbidity with a prevalence inversely proportional to gestational age and is epidemiologically associated with adverse short- and long-term outcomes.^3^ Bronchopulmonary dysplasia (BPD) is childhood's most prevalent chronic lung disease.^4^ Premature neonates who develop this condition in the neonatal period have worse lung function in adulthood.^5^

Different interventions may positively impact neonatal respiratory outcomes in the short term, such as antenatal and postnatal corticotherapy and caffeine.^6,7^ Nevertheless, multiple cycles of antenatal corticotherapy and postnatal corticosteroid use may adversely affect the individual's long-term development.^8,9^ In the neonatal period, caffeine promotes a reduction in the frequency and severity of apneic episodes and reduces the need for mechanical ventilation, but the long-term effects of caffeine are still poorly defined.^6,10^

Adenosine is an essential endogenous sleep inducer and gradually accumulates during wakefulness, inducing physiological sleep.^11^ Caffeine is a competitive inhibitor of adenosine receptors, mainly A1 and A2A, and prolongs latency, reduces total time, and decreases sleep efficiency in adults.^12,13^

Thus, there is concern that prolonged inhibition of adenosine receptors by caffeine in the premature brain may affect the development of neuronal systems that control sleep, with long-term repercussions.^13^ In the neonatal period, caffeine did not alter the sleep pattern of premature newborns.^14^ In the long term, the effects of caffeine administered in the neonatal period on the sleep of these children are not completely elucidated.^15–18^ Twin pregnancy, gender, and intrauterine growth restriction are other factors that may modulate the effects of prematurity and caffeine in long-term sleep development.^19–21^

Thus, this study aimed to evaluate whether caffeine used as a treatment for apnea of prematurity alters late sleep habits and is associated with the presence of obstructive sleep apnea in children aged 5–10 years.

## METHOD

All procedures were submitted and approved by the Research Ethics Committee of the Universidade Federal de São Paulo/Hospital São Paulo (UNIFESP/HSP) through the Plataforma Brasil CAAE n° 33316520.5.0000.5505 (CEP/UNIFESP n° 0630/2020). A convenience sample was used among patients adhering to follow-up at the UNIFESP Premature Outpatient Clinic during their usual consultation period. Due to sanitary restrictions, obtaining the control sample was carried out through the remote application of questionnaires (using the Google Forms platform), maintaining the pairing of cases and controls according to gender and age. All procedures were started after CEP/UNIFESP approval.

Inclusion criteria were as follows:

- Children aged 5–10 years.
- Preterm infants born between 23 and 36 weeks and 6 days of gestational age at the Hospital São Paulo or at the hospitals affiliated to Associação Paulista para o Desenvolvimento da Medicina (Hospital Estadual de Diadema and Hospital Municipal Vereador José Storopolli) who are followed at the Premature Outpatient Clinic of EPM/UNIFESP.
- Any children born at term who met the inclusion and exclusion criteria and that the caregivers validly answered the Google Forms.

Exclusion criteria included children who have started puberty, with a diagnosis of congenital malformations, and children born at term who have been admitted to the neonatal intensive care unit.

After recruitment, children were classified into three experimental groups:

- PCG (preterm caffeine): born preterm and who received caffeine in the neonatal period;
- PNCG (preterm without caffeine): preterm infants born and who did not receive caffeine in the neonatal period; and
- TG (term): born at term.

Children's guardians answered the Children's Sleep Habits (CSHQ)^22^ and the Pediatric Screening Instrument for Obstructive Sleep Apnea (PosaST)^23^ questionnaires. The CSHQ includes questions about sleeping and waking times on weekdays and weekends and total daily sleep time, and a score above 41 indicates possible sleep disturbances. In the PosaST, a score greater than 2.72 indicates obstructive apnea.

The global scores were calculated as described in the Portuguese-language versions of the questionnaires. The validation process of the CSHQ in the Portuguese language generated five subscales related to sleep-disordered breathing, night awakening and parasomnias, sleep duration, difficulty in settling to sleep alone/sleep anxiety, and daytime somnolence that were individually scored and analyzed.

The perinatal factors that may interfere with the children's sleep were surveyed to complement the data in this study. To this end, those responsible for children born at term completed, using Google Forms, a data form containing information regarding gestational history, clinical history, and obstetric and neonatal complications. In the case of preterm infants, perinatal information was obtained by consulting the medical records of these children, which were made available at the Premature Outpatient Clinic of UNIFESP.

The Jamovi software (JAMOVI version 2.3.21.0, SAS Institute Inc., Cary, NC, USA) was used for statistical analysis. The descriptive analysis of quantitative perinatal variables was described as mean and standard deviation and as frequencies in the case of qualitative variables. ANOVA and the Kruskal-Wallis test were used to investigate the differences in continuous variables between groups after normality and homogeneity were verified through the Shapiro-Wilk and Levene tests, respectively. In ANOVA, Tukey's post hoc test was used when indicated. Dwass-Steel-Critchlow-Fligner pairwise comparisons were performed after Kruskal-Wallis test when appropriate. Qui-squared and Fisher's exact tests were used for the analysis of categorical variables, and the post hoc adjusted residual analysis was used to identify the associations.

For the investigation of perinatal factors that may influence the scores in the two questionnaires, linear regression was used. In the linear regression analyses, the dependent variables were established as the score of the CSHQ questionnaire, the total time of daily sleep and the time of waking up during weekdays, and the perinatal information was selected as an independent variable. Data normality was verified employing the Shapiro-Wilk test, multicollinearity employing the Durbin Watson test, and tolerance and homoscedasticity by Cook's distance. The investigation of perinatal factors that could impact these outcomes included age, intrauterine growth restriction, twin birth, gender, use of antenatal corticoids, need for resuscitation in the delivery room, diagnosis of bronchopulmonary dysplasia and periventricular hemorrhage, and the postnatal use of caffeine or corticoids. In all analyses, the level of significance used was 0.05.

## RESULTS

Data collection for preterm infants occurred between October 2020 and February 2021, and that for term infants occurred in May and June 2021. A total of 12 children were excluded from the analysis according to one of the following factors: perinatal data could not be obtained (n=3), preterm births that were not followed up in the Outpatient Clinic (n=5), and outside the age range between 5 and 10 years (n=4). The final sample, therefore, was composed of 45 children from the TG group, 22 from the PCG group, and 20 from the PNCG group, totaling 87 children. The demographic, maternal, and clinical data of the total population studied are presented in Table 1, and the perinatal data of the prematurely born children are given in Table 2.

**Table 1:** Description of demographic and clinical data and maternal data of the 87 study participants.

Variables		PCG (n=22; 25%)	PNCG (n=20; 23%)	TG (n=45; 52%)	p-value
Demographic data					
	Sex: female	12 (54.54)	15 (75)	25 (55.56)	0.285
	Age, years*	7.36±1.62	6.8±1.40	7.00±1.51	0.486
Clinical data					
	Multiple gestation	3 (13.64)	2 (10)	0 (0)	0.029
	Antenatal corticosteroid	12 (54.54)	7 (35)	2 (4.45)	<0.001
	Cesarean section	16 (72.73)	15 (75)	31 (68.89)	0.868
	Gestational age considered (days)	205±16	234±10	281±8	<0.001
	Weight, g	1117±288	1633±273	3260±427	<0.001
	Length, cm	36±3.13	40±2.07	49.00±2.17	<0.001
Weight adjustment					<0.001
	SGA	3 (13.64)	11 (55)	4 (8.89)	
	AGA	19 (86.36)	9 (45)	34 (75.56)	
	LGA	0 (0)	0 (0)	7 (15.56)	
	APGAR 1st min	6.68±1.67	7.75±1.52	8.90±1.14	<0.001
	APGAR 5th min	8.68±1.09	9±0.79	9.79±0.41	<0.001
	Resuscitation in the delivery room	10 (45.45)	5 (25)	0 (0)	<0.001
Maternal medical history					
	Systemic arterial hypertension	4 (18.18)	0 (0)	1 (2.22)	0.014
	Diabetes mellitus	1 (4.54)	0 (0)	0 (0)	0.224
	Cardiopathy	0 (0)	0 (0)	0 (0)	–
	Pneumopathy	0 (0)	0 (0)	2 (4.45)	1.0
Obstetric complications					
	Intrauterine growth restriction	1 (4.54)	6 (30)	2 (4.45)	0.007
	Hypertensive disorder of pregnancy	13 (59.09)	7 (35)	3 (6.67)	<0.001
	Gestational diabetes	3 (13.64)	2 (10)	5 (11.11)	0.607
	Chorioamnionitis	0 (0)	0 (0)	0 (0)	–

PCG: preterm caffeine group; PNCG: preterm caffeine-free group; TG: full-term group; SGA: small for gestational age; AGA: adequate for gestational age: LGA: large for gestational age; APGAR: sum of five signs (muscle strength, heartbeat frequency, reflex, breathing, and color), determined in the first 1 and 5 min of life. The sum generates a score ranging from 0 to 10.

* Mean±standard deviation. Statistical analysis of the categorical variables was performed by using Qui-squared test. The post hoc adjusted residual analysis identified significant associations between term gestation and single pregnancies, preterm birth and antenatal corticosteroid administration, SGA and PNCG, TG and least indication of resuscitation in the delivery room, IGR and PNCG, TG, and the absence of hypertensive disorder of pregnancy. Continuous variables were compared by Kruskal-Wallis test that identified differences in the GA (TG>PNCG>PCG), birth weight (TG>PNCG>PCG), birth stature (TG>PNCG>PCG), first minute (TG>PNCG=PCG), and fifth minute (TG>PNCG=PCG) Apgar scores.

**Table 2:** Neonatal morbidities and therapeutic interventions during hospital stay of the 42 preterm infants.

Variables		PCG (n=22; 52%)	PNCG (n=20; 48%)
Respiratory distress syndrome		21 (95.45)	14 (70)
Bronchopulmonary dysplasia		7 (31.82)	1 (5)
Apnea of prematurity		21 (95.45)	1 (5)
Persistent ductus arteriosus		6 (27.27)	1 (5)
Sepsis		7 (31.82)	2 (10)
Meningitis		1 (4.54)	0 (0)
Necrotizing enterocolitis		1 (4.54)	1 (5)
Periventricular hemorrhage		8 (36.36)	2 (10)
Leukomalacia		0 (0)	0 (0)
Retinopathy of prematurity		5 (22.73)	0 (0)
Metabolic bone disease		0 (0)	0 (0)
Mechanical ventilation		17 (77.27)	3 (15)
	Days on mechanical ventilation	14.8±16.7	9±13
Corticosteroid		4 (18.18)	1 (5)
	Days of corticosteroids	5±4	13±0
Diuretics		7 (31.82)	1 (5)
	Days of diuretics	26.4±23.7	–
Caffeine		22 (100)	0 (0)
	Days of caffeine	29.9±14.3	0 (0)
Parenteral nutrition		19 (86.36)	7 (35)
	Days of parenteral nutrition	21.5±30.8	11.9±9.28
Full enteral nutrition, days		16.5±5.72	10.5±3.54
Weight loss, %		-8.32±4.78	-7.95±4.55
Day for weight recovery		10.3±5.81	8.88±3.82
Lemght of hospital stay in days		67.6±39.2	42.6±48.7
Corrected gestational age at hospital discharge in weeks		39 2/7±5 2/7	39 2/7±6 3/7

PCG: preterm caffeine group; PNCG: preterm non-caffeine group. Mean±standard deviation or number (%) as appropriate.

**Figure 1:**
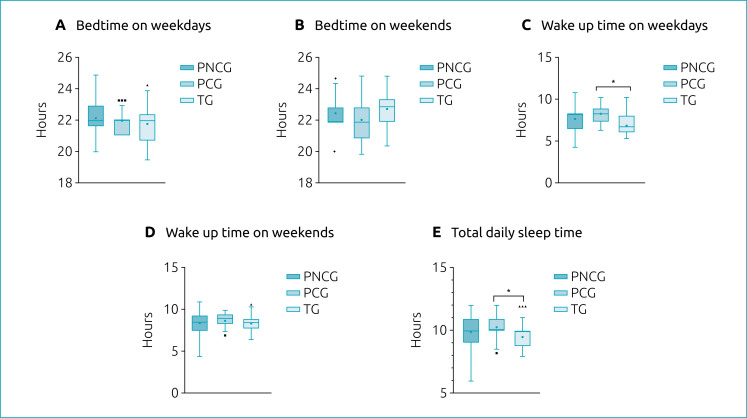
Times of the children's sleep routine. (A) Sleeping hours on weekdays; (B) sleeping hours on weekends; (C) waking hours on weekdays; (D) waking hours on weekends; and (E) total daily sleep time. *p<0.05; PCG: preterm caffeine group; PNCG: preterm non-caffeine group; TG: term group. The triangles and squares represent outliers.

The analysis of variance identified a difference in the wake-up time between the groups (F_(2,83)_=8.04; p=0.001). Tukey's post hoc test identified that the PCG group woke up significantly later on weekdays than the TG group (09h±00h58 and 07h43±1h15, respectively, p=0.022). A difference was also found in total sleep time between the groups (F_(2,84)_=3.74; p=0.028). Tukey's post hoc test showed that children in the PCG group had significantly longer total daily sleep time compared to children in the TG group (10h24±1h08 and 09h29±1h08, respectively, p≤0.001). In the other variables, no significant differences were observed (Figure 1).

No statistically significant differences were identified in the CSHQ questionnaire scores between the PNCG, PCG, and TG groups (45.90±7.14, 46.70±8.97, and 46.00±6.85, respectively, F_(2,84)_=0.102; p=0.904) or in the PosaST questionnaire (1.59±1.83, 1.19±1.54, and 1.00±1.35, respectively, F_(2,84)_=0.215; p=0.807). The percentage of individuals presenting results above the cutoff limit in the two questionnaires was similar in the three groups; χ^2^_(2)_=0.478; p=0.787 for CSHQ; and χ^2^_(2)_=4.74; p=0.129 for PosaST, respectively (Table 3).

**Table 3:** Number and percentage of individuals with results above the cutoff limit in CSHQ and PosaST questionnaires.

Groups	Total number of individuals	Number (%) above the cutoff point in CSHQ	Number (%) above the cutoff point in PosaST
PNCG	20	14 (70)	5 (25)
PCG	22	15 (68)	2 (9)
TG	45	34 (75)	3 (6.6)

PCG: preterm caffeine group; PNCG: preterm caffeine-free group; TG: full-term group.

Analyzing the subscales of the Portuguese version of the CSHQ questionnaire, there were differences in the scores in the sleep duration subscale (H_(2)_=7.88; p=0.019). Comparison between pairs showed that the PCG group had a significantly higher mean score than the TG group (9.67±1.43 and 8.56±1.65, respectively; W=3.86; p=0.017). No significant differences were found between the groups’ sleep-disordered breathing, night awakening and parasomnias, difficulty settling to sleep alone/sleep anxiety, and daytime somnolence scores.

In linear regression analyses, the models adjusted for total daily sleep time, wake-up time during the week, and CSHQ score showed correlation coefficients of R^2^=0.198, R^2^=0.368, and R^2^=0.194, respectively. Only twinship (β=1.9, p=0.007) was related to waking hours during the week. That is, children born from twin gestation woke up, on average, 1 h and 54 min later than those born from single gestation.

## DISCUSSION

The relationship between prematurity and poorer sleep quality in children has not yet been widely studied and elucidated.^17,18^ Some studies point out that there is no association between these factors; however, others state that prematurity increases the probability of the late development of sleep disorders and their consequences such as night awakening and difficulty falling asleep.^15–18^

When comparing the mean scores obtained in the CSHQ questionnaire, there was no significant difference between the three groups studied. This fact contradicts a study that used the same questionnaire and found that the mean score of preterm infants was significantly higher (47.6±9.2) when compared to term infants (44.8±7.6).^15^ However, the three groups had a mean questionnaire score above the cutoff point (>41), which was determined to indicate the presence of sleep disorders. This finding agrees with a study comparing sleep in preschool children born at term and preterm, in which 70.8% of the children born preterm and 60.7% born at term also had scores above the cutoff point.^15^ However, it is noteworthy that a study comparing data obtained with CSHQ and actigraphy reported that for the sleeping hours during the week, averages of 21h51±00h34 and 22h25±00h35; for the wake-up time during the week, 07h52±00h35 and 08h00±00h37; and for the total daily sleep time, 10h28±1h06 and 08h03±00h38 according to parents information and actigraphy, respectively.^24^ Another study evaluating only preterm infants exposed and not exposed to caffeine in the neonatal period by questionnaires, actigraphy, and polysomnography did not find any differences in objective and subjective sleep parameters in school-age individuals. In the polysomnography single-night evaluation, preterm infants exposed to caffeine presented longer total sleep time but equal sleep efficiency. The difference in sleep duration was not confirmed by the 2-week actigraphy evaluation, which was considered more accurate by the authors.^25^ Despite the variation in these results, the questionnaire has been widely used and represents a viable instrument for evaluating children's sleep habits.

When evaluating the three groups, the results of our study showed that the PCG group presented total sleep time significantly longer than the TG group. This finding contradicts a study carried out with adolescents between 16 and 19 years of age born preterm and full-term, which found, employing actigraphy, that prematurity was associated with earlier sleep (approximately 22 min) after adjusting for age, race, gender, maternal education, and family income.^26^ Analyses also showed that a significantly higher proportion of adolescents born prematurely reported regular bedtimes compared with their term peers (60.2 vs. 43.6%). Regular sleeping and waking times are important factors related to sleep quality, and the results found by these authors may indicate that preterm infants establish an adequate sleep pattern. Interestingly, the authors showed that among the participants with a regular bedtime, children's bedtime was more frequently set by the parents for the preterm group compared to the term group (30.8 vs. 16.3%).^26^

PCG's increased scores in the CSHQ sleep duration subscale compared to TG disagree with the results obtained from maternal information. When reviewing the questions that integrated the subscale, some analyze children's behavior at sleep time (questions 1, 2, 6, and 25), and others infer sleep duration qualitatively (questions 9, 10, and 11). The answer to the behavior questions may inflate the score, as we accurately describe increased sleep time in our population. Considering the nature of the questions combined in this factor in the validation process, “sleep duration” does not seem to be an adequate denomination and should be considered carefully.

In our sample, children in the PCG group woke up significantly later than the TG group on weekdays, according to maternal reports. However, this result does not confirm the findings of a study carried out with 2-year-old children that also used maternal reports and concluded that there are no differences between preterm infants who woke up, on average, at 7h45 and term infants who woke up, on average, at 7h40.^27^

It is essential to highlight that the two studies that presented different findings from those found in the present study refer to different age groups; therefore, a complete evaluation involving different ages may be instrumental in understanding how sleep can be impacted throughout the life of preterm infants. Likewise, cultural differences and different methodological approaches must be considered when comparing results.

In our study, twinship seems to be a factor that impacted the wake-up time during the week of preterm births, which happened later (β=1.9; p=0.007). Children born from twin gestation woke up on average 1h54 later than those not born from twin gestation. The effect of twin pregnancy has already been evaluated in a study involving 197 premature children aged 5–12 years employing ambulatory polysomnography, in which perinatal risk factors associated with obstructive sleep apnea syndrome (OSAS) were investigated. In this study, the authors concluded that multiple gestations were positively associated with OSAS; however, no changes in waking or sleeping times were reported.^19^ Despite using a convenience sample in the present study, we are confident in our result. We carried out an a posteriori analysis of the sample size. Considering that the linear regression included 10 predictors and presented an R^2^=0.368 and that 87 individuals were included in the analysis with a significance level of 0.05, the calculated power was 0.999.

Comparing the mean scores of the PosaST questionnaire, no significant differences were observed between the groups. This finding does not agree with a study carried out with 282 children born at term and preterm in preschool age, which showed that OSAS symptoms were present in 60.6% of the children born preterm and in 29.7% of those born at term.^15^ However, it is important to note that one study pointed out that the sensitivity of the questionnaire to diagnose mild degrees of OSAS using the cutoff point of ≥2.72 exhibited a sensitivity of only 17% despite a specificity of 95%. In contrast, for moderate and severe cases, the sensitivity was 46%.^28^ The PosaST has been widely used and represents an instrument with high potential in OSAS suspicion to be used in the pediatric population since the gold standard test for the disease, polysomnography, is costly and difficult to access.^23^

We considered questionnaires a limitation of this study since the investigation of sleep habits, and the presence of obstructive apnea was carried out only by subjective methods. The alternative would be to use objective methods such as actigraphy and polysomnography together with the questionnaires, but, as previously mentioned, these are more complex methods. Therefore, we emphasize that questionnaires are a reliable option for screening for sleep disorders in children and can provide important information that will help in clinical management.^23^

The COVID-19 pandemic may have impacted the results of the present study, causing abrupt changes in the homes resulting in the absence of routine and changes in the schedules of the activities of children and their caregivers.^29^ However, because our data collection was conducted exclusively during the pandemic, there is no way to determine the influence of the pandemic on outcomes because we have no prior data. In addition, data collection for the preterm and full-term groups occurred at different times of the year, and the pandemic scenario may have interfered with the results. Face-to-face classes were suspended in São Paulo throughout the children's recruitment period, considering the lockdown and current health recommendations. In this way, we can assume that none of the participating children were attending schools.

The demographic characteristics of the preterm exposed and not exposed to caffeine were also different, which could suggest that the groups could not be compared. In a Norwegian study that investigated prenatal and neonatal factors that could predict sleep problems in 221 preterm-born 11-year-old children, only maternal smoking was associated with an increased risk of snoring, and periventricular hemorrhage and intrauterine growth restriction were associated with difficulty falling asleep or frequent awakenings.^30^ These two factors were incorporated into our linear regression model.

Therefore, we conclude that the information found in the present study indicates that caffeine used in the neonatal period as a treatment for apnea of prematurity does not impair sleep in children. Increased sleep time in preterm-born infants in the PCG group may be related to the long-term upregulation of adenosine receptors in the developing neonatal brain exposed to caffeine, but this hypothesis has to be confirmed in future studies.

## Data Availability

The database that originated the article is available with the corresponding author.
